# The Effectiveness of Exercise Programs in Adolescents with Thoracic Kyphosis: A Narrative Review

**DOI:** 10.3390/healthcare12151503

**Published:** 2024-07-29

**Authors:** Seoyon Yang, You Gyoung Yi, Min Cheol Chang

**Affiliations:** 1 Department of Rehabilitation Medicine, College of Medicine, Ewha Woman’s University, Seoul 03760, Republic of Korea; seoyonyang@gmail.com (S.Y.); lyk861124@gmail.com (Y.G.Y.); 2 Department of Physical Medicine and Rehabilitation, College of Medicine, Yeungnam University, Daegu 42415, Republic of Korea

**Keywords:** exercise, rehabilitation, thoracic kyphosis

## Abstract

Thoracic kyphosis is a common postural problem affecting over 20% of adolescents. This condition can contribute to various spinal problems, leading to a decreased ability to perform daily activities, reduced quality of life, and impaired pulmonary function. This review aimed to investigate the effectiveness of exercise programs in adolescents with thoracic kyphosis. We systematically searched the PubMed, Embase, Cochrane Library, and Scopus databases for articles relevant to adolescents with thoracic kyphosis that had been published up to 14 May 2024. Our inclusion criteria focused on studies investigating the effects of exercise on improving thoracic kyphosis. A total of 1883 articles was identified using the search terms. After the titles and abstracts had been screened, 1868 articles were found not to meet our inclusion criteria and were excluded. The remaining 15 articles were then assessed for eligibility. Finally, only seven studies were included in this systematic review. Exercises targeting the entire spinal curvature demonstrated efficacy in enhancing the strength and function of the cervical, thoracic, lumbar, and pelvic muscles, resulting in a corrective effect on thoracic kyphosis in adolescents. Consequently, exercise programs have emerged as potentially beneficial treatment approaches to improve poor posture and reduce adolescent thoracic kyphosis.

## 1. Introduction

Thoracic kyphosis is a condition that frequently begins during adolescence, which is a period of growth, and can progress further as one ages [1].

It is a common postural problem observed in more than one out of five adolescents [2]. Adolescents have recently become more susceptible to postural disorders, such as thoracic kyphosis. This is mostly likely due to the increased use of digital devices, such as smartphones and personal computers (PCs) [3], which has caused them to adopt improper postures that further worsen their structural deformities.

In adolescents, the normal thoracic angle is 20°–40°. The Cobb angle is used to measure kyphosis by drawing one line along the upper epiphyseal plate of T5 and another line along the lower epiphyseal plate of T12. Thoracic kyphosis is diagnosed when the kyphotic curve exceeds 40° in the thoracic spine [4,5]. In rapidly growing adolescents with thoracic kyphosis, abnormal thoracic-spine flexion can impede the development of the internal organs and interfere with normal respiratory function [6]. Deviations from ideal spinal curvatures that exceed normal limits can increase intradiscal pressure and alter the load distribution on the spine. This can increase the risk of developing injuries, such as back pain, disc herniations, muscle strains, and ligament and tendon injuries [7]. It can also result in a decreased ability to perform daily activities, reduce quality of life, and impair pulmonary function [8]. Additionally, as kyphosis progresses, it may lead to gait disturbances, body sway, and poor balance, which can increase the risk of falls [9].

The progression of thoracic kyphosis can lead to a rapid degeneration of the spinal column, and it can also affect the adolescents’ posture and appearance, potentially affecting their physical and psychological health. Therefore, efforts should be made to control the progression of thoracic kyphosis and treat it as early as possible. Common treatments for kyphosis include pain medications, bracing, exercises, and surgery [10,11]. Previous studies have reported that exercise improves kyphosis in adults [12,13]; however, the benefit of exercise in adolescents with thoracic kyphosis has not yet been thoroughly investigated. Therefore, this review aimed to evaluate the effectiveness of exercise programs in adolescents with thoracic kyphosis.

## 2. Methods

We conducted a narrative review of studies investigating the effects of exercise on adolescents with thoracic kyphosis.

### 2.1. Search Strategy

We researched relevant articles using the PubMed, Embase, Cochrane Library, and SCOPUS databases to search for articles published through 14 May 2024. The search used the keywords “thoracic kyphosis” and “exercise”.

### 2.2. Study Selection

Any type of study that investigated the effects of exercise on adolescents with thoracic kyphosis was included in the search. The primary outcome of interest was improvement in the thoracic-curve angle. The inclusion criteria for article selection were as follows: (1) studies involving adolescents with thoracic kyphosis, (2) application of exercise programs to correct thoracic kyphosis, and (3) measurements of the thoracic-curve angle before and after interventions. The exclusion criteria were as follows: (1) studies not related to adolescents with kyphosis; (2) animal studies, case reports, reviews, commentaries, and letters; and (3) studies with insufficient or unreported outcomes. Two independent reviewers (S.Y.Y. and M.C.C.) screened the articles based on their titles and abstracts. Subsequently, full-text assessments were conducted before the final inclusion. Any disagreements were resolved by consensus among the reviewers, with the input of a third reviewer (Y.J.C.) if necessary.

### 2.3. Data Extraction

Two reviewers (S.Y.Y. and M.C.C.) extracted data independently using a standard data-collection form. The following data were recorded from each eligible article using a table: (1) first author’s name, (2) year of publication, (3) study design, (4) number of participants, (5) age range, (6) details of exercise control groups, and (7) treatment duration.

## 3. Results

**Figure 1:**
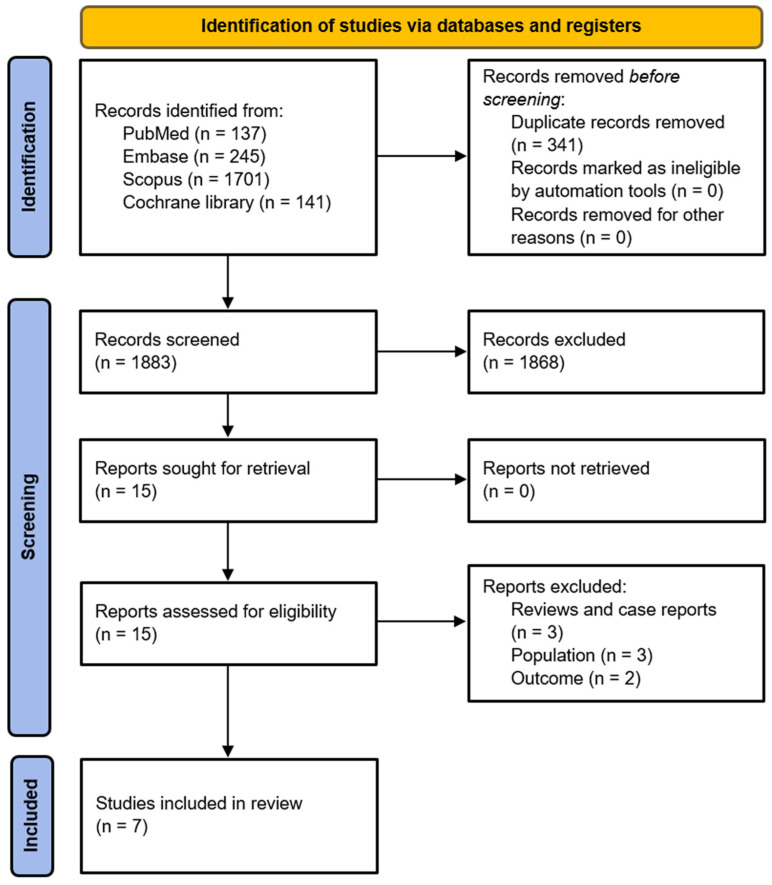
Flow diagram of the study-selection process.

Following the initial search, 1883 articles were identified using the search terms. After reading the titles and abstracts, the articles that were not relevant to thoracic kyphosis or exercise, did not measure the thoracic-curve angle, or had participants who were not adolescents were excluded. We also excluded animal studies, case reports, reviews, commentaries, letters, and studies with insufficient or unreported outcomes. As a result, a total of 1868 articles were excluded. Subsequently, the remaining 15 articles were assessed for eligibility, and 8 articles were excluded because three were reviews of the literature or case reports, three were not focused on adolescents with thoracic kyphosis, and two did not provide sufficient outcome data. Ultimately, seven articles investigating the effects of exercise on adolescents with thoracic kyphosis were included in this review (Figure 1). All studies were randomized controlled trials [7,14,15,16,17,18,19]. Three studies used the Cobb angle to measure thoracic kyphosis [15,16,18], whereas the other studies used different measuring tools, such as an inclinometer [17] or the Spinal Mouse system [7,14,19]. Studies applied different types of exercises, including stretching, strengthening, Pilates, and the Schroth method. The characteristics of these studies are summarized in Table 1.

In a 2018 study, Feng et al. reported the beneficial effects of a functional exercise program in adolescents with postural kyphosis [19]. This study included 164 adolescents diagnosed with thoracic kyphosis, who were divided into the corrective-functional-exercise group (n = 81) and the control group (n = 83). The functional exercise program comprised exercises targeting the cervical, thoracic, lumbar, and pelvic regions that were specifically designed to correct thoracic kyphosis. The control group followed a standard exercise program involving abdominal curls, push-ups, squats, and a 50-m run. Both groups attended 8 weeks of exercise sessions, with two weekly sessions lasting 15–20 min each. The thoracic kyphotic angle (TKA) was measured using the Spinal Mouse system (Idiag, Fehraltorf, Switzerland). Following the exercise programs, significant changes in the TKA (approximately 9°), sacral angle, and thoracic range of motion (ROM) were observed in the functional-exercise group compared with the control group. The functional exercise program, which targeted the entire spinal curvature, led to improvements in the cervical, thoracic, and lumbar ROM and muscle strength and function, thereby positively affecting thoracic kyphosis in adolescents.

In 2019, Bezalel et al. conducted a randomized controlled trial (RCT) to investigate the effect of exercise on adolescents with Scheuermann’s disease, also known as juvenile kyphosis, which is a common condition characterized by thoracic spine kyphosis [18]. This study included 50 adolescents aged 10–17 years. The exercise group (n = 25) performed five classic Schroth therapy exercises, whereas the control group (n = 25) performed five classic anti-gravitation exercises. The Schroth therapy program involved corrective reeducation of the neuromuscular system, therapeutic exercises, and special breathing techniques. The control group performed five conventional exercises. The participants in both groups received one treatment session weekly for 12 months. Significant improvements in kyphotic deformity, measured using an inclinometer, were found between the two groups (exercise group, 10.54° ± 7.65° vs. control group, 4.09° ± 6.71°). Moreover, the exercise group demonstrated a more significant improvement in thoracic kyphosis, as measured by the thoracic Cobb angle (8.78° ± 8.38°), compared to the control group (3.57° ± 7.59°). This study concluded that the Schroth therapy effectively improved thoracic kyphosis and reduced pain in adolescents with Scheuermann’s disease.

In 2020, González-Gálvez et al. conducted a study involving 236 adolescents aged 12–17 [7]. The exercise group (n = 118) participated in Pilates exercises for 9 months (two sessions per week, 15 min/session), while the control group (n = 118) did not engage in any exercise regimen. The sagittal spinal curvatures were assessed during relaxed standing using the Spinal Mouse system. The Pilates program comprised three phases categorized by exercise difficulty. These exercises, including half roll-up, one-leg stretch, swimming, mid-back bending, and shoulder bridge, strengthen the abdominal and back muscles and promote trunk stability. After 9 months, the exercise group exhibited increased hamstring extensibility and decreased lumbar curvature without increased thoracic curvature. By contrast, the control group showed worsening thoracic kyphosis while standing. Performing the Pilates exercises for 9 months did not result in a significant decrease in the thoracic curvature; however, it effectively halted its progression. 

In 2022, Elpeze et al. investigated the effectiveness of exercise in improving thoracic kyphosis [17]. The study included 62 patients (10–18 years of age) with kyphosis angles ≥ 50°. The patients were assigned to one of three groups: a comprehensive corrective exercise program (CCEP) group (n = 21), a thoracic exercise program (TEP) group (n = 22), or a control group that was assigned no exercise (n = 19). The thoracic kyphosis angle was measured using a smartphone inclinometer (Samsung, Clinometer Version 2.4) and a flexible ruler (Flexicurve) based on the anatomical landmarks (T1–T12). The exercise group engaged in exercise programs three times a week for 12 weeks, with each session lasting 40–50 min. CCEP involved corrective exercises (chin-tuck exercises, stretching of the neck extensor and pectoral muscles, arm and leg lifts, cat-camel stretches, and bridging exercises) and postural perception training (PPT). During the PPT, the participants were instructed to recognize their habitual postures, differentiate between poor and good postures in sitting and standing positions before a mirror, and adjust their positions to achieve good posture during the training sessions. The PPT assessment involved the patients reporting the number of times they recognized and corrected their bad posture weekly, and then the average was recorded. The TEP included stretching of the pectoral muscles; thoracic self-mobilization; T, Y, W, and I; and cat-camel exercises. The CCEP and TEP groups showed reductions in kyphosis angle compared to the control group, with a more significant decrease observed in the CCEP group (8.93°) than in the TEP group (4.33°). As measured by the Romberg index, only the CCEP group showed improvements in PPT and balance, whereas no changes were observed in the TEP or control groups. This study underscored the efficacy of combining corrective exercises with PPT to correct thoracic kyphosis.

In the same year, Seo et al. conducted a study to assess the impact of an exercise program on 60 adolescents aged 10–19 years who had been diagnosed with postural kyphosis, which was defined as a Cobb’s angle ranging from 30° to 45° [16]. The adolescents were divided into the exercise (n = 25) and control (n = 26) groups. The exercise program consisted of stretching the major joints and muscle groups, as well as neck and trunk exercises, such as chin-ins, trunk extensions, arm and leg raises, and Schroth method exercises. This program was administered by an exercise specialist over 12 weeks, with sessions lasting 60 min and conducted three times a week. The results indicated that the exercise group exhibited significant improvements in the Cobb’s angle (pre-intervention, 16.23 ± 1.81°; post-intervention, 10.77 ± 3.35°, *p* < 0.00) and forward head angle (pre-intervention, 38.22 ± 3.76°; post-intervention, 26.95 ± 5.03°, *p* < 0.00) as compared to the control group. These findings supported the effectiveness of exercise interventions for improving spinal alignment and balance in adolescents with postural kyphosis.

In 2023, Gheitasi et al. conducted a study involving 180 adolescents aged 11–16 years with thoracic hyperkyphosis at angles of 45° or higher, as measured using the Cobb method [15]. The participants were divided into three groups: corrective exercises plus bracing (60 participants), bracing (60 participants), and control (60 participants). The Cobb angles of the thoracic curves were measured at baseline and 24 weeks post-intervention. The exercise program lasted for 24 weeks, with sessions lasting 60 min each and conducted three times a week. The patients were provided with customized Milwaukee braces. The exercise program comprised three phases. The initial phase involved education on appropriate and symmetric physical conditioning, stretching exercises, isometric exercises, and core stability exercises, such as pelvic tilt, W shoulder exercises, and seated rows. The improvement phase focused on transitioning from static to dynamic postural-correctional states (including Y, W, and T exercises; prone shoulder flexion; and upper-back extension exercises). The maintenance phase spanned 3 weeks and aimed to sustain training adaptations by practicing optimal posture during various activities such as sitting, standing, and walking. The results indicated a significant decrease in the mean Cobb angle in the corrective-exercise-plus-bracing group but an increase in the mean Cobb angle in the control group after 24 weeks. The between-group analysis revealed significant differences among all groups, indicating that combining corrective exercises and bracing led to better outcomes in the Cobb angle than bracing alone or no intervention.

In the same year, González-Gálvez et al. enrolled 103 adolescents with thoracic hyperkyphosis, defined as an angle greater than 40°, who were not receiving specific treatment [14]. The forty-nine participants in the experimental group performed a Pilates exercise program for 38 weeks (two sessions/week, 15 min/session). The Pilates exercise program consisted of stretching exercises and exercises focusing on strengthening the rectus abdominis, oblique, and paravertebral muscles, such as half roll-ups, crisscrosses, front supports, swimming, shoulder bridges, and one-leg stretches. The forty-eight participants in the control group did not participate in any exercise program or regular physical education lessons. Using the Spinal Mouse system, the sagittal spinal curve was assessed in sit-and-reach and relaxed standing positions. The participants who had thoracic curves > 40° in the relaxed standing position were defined as having hyperkyphosis. The participants who underwent the Pilates exercise program showed a significant change in the thoracic curve in the relaxed standing position as compared to the controls. The exercise group showed a final hyperkyphosis rate of 53% (n = 26), in contrast to the rate of 77% (n = 26) in the control group. After nine months in the Pilates exercise program, the thoracic kyphosis improved and the participants had angles close to the normal value (42°). This study suggests that adolescents with thoracic hyperkyphosis benefited from decreased thoracic kyphosis after 9 months of performing the Pilates exercise program.

In summary, all seven studies investigating the efficacy of exercise interventions for thoracic kyphosis concluded that various exercise programs, including corrective functional exercise, postural education, Schroth exercise, and Pilates exercise, can promote healthier sagittal spinal disposition by improving spinal stability and trunk strength in adolescents with kyphosis.

## 4. Discussion

This review included studies that examined the differences in the thoracic-curve angle before and after exercise programs in adolescents with thoracic kyphosis to determine whether the thoracic curve was actually affected by exercise programs. We confined our search terms to “thoracic kyphosis” and “exercise” to include studies investigating the effect of exercise on thoracic kyphosis, regardless of age, and then hand-searched for and identified those studies conducted on adolescents. The findings from the studies indicated that exercise programs can decrease thoracic curvature in adolescents with thoracic kyphosis. Abnormal spinal alignment can increase biomechanical stress on the thoracic spine and impair the neuromuscular, sensory, and musculoskeletal systems [20]. The progression of thoracic kyphosis can cause muscle weakness, pain, decreased range of motion in the joints, dysfunction, misalignment of the entire spine, increased forward head posture, decreased lumbar lordosis, and increased scapula rotation [21]. Additionally, it shifts the body’s center of gravity backward, causing imbalance, uneven foot weight distribution, and an increased risk of falling [22]. Therefore, interventions to correct abnormal spinal alignment are crucial for enhancing spinal stability, preserving healthy sagittal spinal balance, and mitigating the progression of thoracic-curve deviation.

Based on the results of the studies included in this review, exercise programs appear to be effective in correcting thoracic kyphosis in adolescents. For adolescents with a thoracic curve of 40° or more, it seems advisable to implement exercise programs as early as possible to prevent further progression of the curvature. All included studies applied both stretching and strengthening exercises in their programs. These regimens incorporated various exercises, such as cat-like stretches, pectoral-muscle stretches, thoracic and pelvic ROM exercises, and core-muscle-strengthening exercises. Although a recent systematic review suggested that strengthening exercises might be more effective than stretching exercises for the correction of thoracic curves [13], it appears that combining both types of exercises is essential. This combined approach of stretching and strengthening exercises seems to be more effective in improving hyperkyphosis than using either type of exercise alone. Of the seven studies included in this review, three demonstrated the effects of exercise over a period of 6 months or more [7,14,18], suggesting that exercise may have long-term benefits for correcting thoracic kyphosis.

The findings of the included studies suggested that corrective functional exercise programs, which take a holistic approach, are more effective in addressing thoracic kyphosis than conventional exercise alone. In general, the corrective exercise programs targeting the thoracic region and the cervical, thoracic, lumbar, and pelvic regions seem to yield superior outcomes in treating thoracic kyphosis compared with the programs focusing solely on the thoracic vertebrae. Thoracic kyphosis often correlates with weakness in the neck flexors, paravertebral muscles, and abdominal muscles. Also, it contributes to increased lordosis in the cervical spine, primarily due to the shortening of the pectoral muscles and spinal extensor muscles [23]. When designing exercise programs for adolescents with thoracic kyphosis, it is essential to incorporate exercises targeting the muscles and the joints throughout the head, neck, trunk, and upper limbs (including the cervical, pectoral, thoracic, and lumbar muscles) and the ranges of motion to enhance muscle flexibility, improve proprioception, and strengthen muscles weakened by spinal asymmetry. These exercises involve strengthening the thoracic or lumbar spinal extensor muscles, cervical retractors, pectoral muscles, or shoulder retractors. Exercise programs that correct the misalignment of the spinal curvature and restore the balance between the tightened and weakened muscles are crucial for improving kyphotic posture [16]. Such programs have the potential to realign the spine and enable the body to maintain proper spinal orientation, even when it is subjected to static and dynamic loads [19].

Two studies included in this review highlighted the benefits of the Pilates exercise program for adolescents with thoracic kyphosis [7,14]. The Pilates exercise program is frequently used to promote a healthy sagittal spine and has been shown to positively affect sagittal spinal posture in adolescents with postural issues [14]. Emphasizing the stretching of the tight muscles and strengthening of both the core and weakened muscles, the Pilates method also targets the entire sagittal spinal curvature rather than focusing solely on the thoracic paravertebral structures [13]. These exercises have been demonstrated to positively affect the sagittal balance of the spine, resulting in an overall improvement in thoracic kyphosis [7,14].

Two studies incorporated the Schroth exercises into their programs and suggested that Schroth therapy could effectively prevent deterioration and reduce the thoracic-angle curve in adolescents with thoracic kyphosis [16,18]. Schroth therapy involves specific breathing patterns and correction of the kyphotic posture through proprioceptive and exteroceptive stimulation and mirror control in the sagittal plane. These exercises encompass a combination of forces that are exerted externally on the vertebral column by correcting the three blocks in the sagittal plane in conjunction with the elongation force on the vertebral column. The primary principles of Schroth exercises focus on both passive and active reduction of the kyphotic hump along with stretching of the hamstring and pectoralis muscles. Over time, the patients engage their trunk muscles in corrective active forces and learn to raise themselves as far as possible from a slumped position. After that, they are required to maintain correct posture while performing activities of daily living, leading to a more upright trunk posture [18]. The Schroth exercises are a practical approach to correcting thoracic kyphosis and promoting good posture.

Maintaining good posture also appears to be relevant to improving thoracic kyphosis among adolescents. Thoracic kyphosis in adolescents can be triggered or aggravated by various factors, including reduced physical activity, carrying heavy schoolbags, and sitting or standing for long hours with incorrect posture. Aligning the head with the pelvis, slightly tucking the chin, retracting the shoulders (pulling the shoulder blades together toward the spine), extending the thoracic spine, and maintaining a slight increase in lordosis were suggested as methods to improve posture in a previous study [17]. Postural education aims to improve awareness, which is closely linked to habitual posture. Poor habitual posture during activities of daily living decreases the ability to perceive, sustain, and readjust to a neutral spinal position. It is crucial for adolescents to first become aware of their posture so that they can correct their poor postural habits effectively [17]. Postural education using visual materials demonstrating the ideal spinal alignment while sitting or standing improves the perception of thoracic alignment, mobility, and proprioception. Postural education should be integrated into the treatment programs so that the patients can become aware of and address their poor postures independently in their daily lives. Combining postural education with exercises to improve spinal mobility and endurance is a practical approach to enhancing posture and reducing the angle of thoracic kyphosis in adolescents with this condition.

This review has several limitations that must be acknowledged. First, the sample sizes of the included studies were relatively small. Second, most adolescents included in these studies had mild-to-moderate deformities, and there could be some bias in the patient-selection process. Additionally, the effect of exercise on adolescents with severe hyperkyphosis was not assessed; hence, the generalizability of the findings to a broader population may be limited. Third, the methods used to measure the kyphotic angle varied across studies. Studies utilized inclinometers, the Spinal Mouse system, or X-rays for measurements. X-rays are considered the most accurate measure of spinal curvature; however, due to concerns regarding the radiation exposure associated with X-rays, the Spinal Mouse system is preferred for evaluating thoracic kyphosis [24]. Measuring using different methods can cause technical differences. Despite these limitations, the effect of exercise was investigated before and after the exercise program, and results suggested that exercise may improve kyphosis. It should also be noted that these studies did not mention whether the thoracic-angle curves of patients who had exercised would deteriorate again when they stopped exercising. There is a possibility that the thoracic angle curves may return to their original state when exercise is discontinued. Future research should be conducted to investigate the long-term effects of exercise on thoracic kyphosis.

## 5. Conclusions

This study summarized the effectiveness of exercise programs and postural education for adolescents with thoracic kyphosis. Exercises that target the entire spinal curvature have demonstrated efficacy in enhancing the strength and function of the cervical, thoracic, lumbar, and pelvic muscles, resulting in a corrective effect on thoracic kyphosis. Consequently, exercise programs have emerged as potentially beneficial treatment approaches to improve poor posture and reduce adolescent thoracic kyphosis.

## Figures and Tables

**Table 1:** Characteristics of the included studies.

No.	Author	Year	Study Design	Number of Participants	Age	Type of Exercise	Control Group	Treatment Duration	Evaluation	Results
1	Feng et al. [19]	2018	RCT	164 adolescents (81 functional exercises vs. 83 standard exercises)	Male: 14.2 ± 1.43 vs. 13.5 ± 1.47, Female: 14.0 ± 1.52 vs. 13.6 ± 1.54	Corrective functional exercise program	Standard exercise program (abdominal curls, squats, etc.)	15–20 min, two times/week for eight weeks	Sagittal spinal curve using the Spinal Mouse system	After the exercise programs, significant changes in TKA (approximately 9°), sacral angle, and thoracic ROM were observed in the functional-exercise group compared to the control group.
2	Bezalel et al. [18]	2019	RCT	50 adolescents (25 Schroth exercises vs. 25 conventional exercises)	10–17 yrs (14.52 ± 1.79 vs. 13.39 ± 1.66)	The Schroth therapy exercises	Conventional exercise (five classic anti-gravitation exercises)	One time/week for 12 months	Cobb’s angle and inclinometer	Significant changes in kyphotic deformity were found between the two groups. The exercise group showed more improvement in thoracic kyphosis, as measured with the thoracic Cobb angle, than the control group.
3	González-Gálvez et al. [7]	2020	RCT	236 adolescents (118 exercises vs. 118 no exercise)	12–17 yrs (13.15 ± 1.24)	Pilates exercise program	No exercise (only regular physical education)	15 min two times/week for 9 months	Sagittal spinal curve using Spinal Mouse system	After 9 months, the exercise group showed increased hamstring extensibility, decreased lumbar curvature, and increased thoracic curvature compared to the control group.
4	Elpeze et al. [17]	2022	RCT	62 adolescents (43 exercises vs. 19 no exercise)	10–18 yrs	Comprehensive corrective exercise program (CCEP) vs. thoracic exercise program (TEP)	No exercise	40–50 min, three times/week for 12 weeks	Kyphosis angle using a smartphone inclinometer and a flexible ruler, survey, the Romberg index	The kyphosis angle was reduced in the CCEP and TEP groups but not the control group. Additionally, there was a more significant reduction in the kyphosis angle in the CCEP group (8.93°) compared to the TEP group (4.33°).
5	Seo et al. [16]	2022	RCT	51 adolescents (25 exercises vs. 26 no exercise)	10–19 yrs (17.35 ± 1.23 vs. 17.72 ± 2.13)	Combined exercise program (stretching, neck/back muscle exercises, and Schroth Method exercise)	No exercise	60 min, three times/week, 12 weeks	Cobb’s angle and forward head angle	The exercise group showed significantly improved Cobb’s angle and forward head angle compared to the control group.
6	Gheitasi et al. [15]	2023	RCT	180 adolescents (60 exercises plus bracing vs. 120 bracing or no exercise)	11–16 yrs	Three phases include stretching, isometric, core stability, and functional exercises.	Bracing only or no exercise (waitlist)	60 min, three times/week, 24 weeks	Cobb angle from standing lateral spine radiographs	Combining corrective exercises and bracing resulted in better overall outcomes in the Cobb angle than did bracing alone or no intervention.
7	González-Gálvez et al. [14]	2023	RCT	103 adolescents (51 Pilates vs. 52 no exercise)	13.48 ± 1.23	Pilates program including stretching and strengthening of abdomen and paraspinal muscles	No exercise	15 min, two times/week for nine months	Sagittal spinal curve using Spinal Mouse system	The participants who underwent the Pilates exercise program showed a significant change in the thoracic curve in the relaxed standing position compared to the control group.

CCEP, comprehensive corrective exercise; RCT, randomized controlled trial; ROM, range of motion; TEP, thoracic exercise program; TKA, thoracic kyphotic angle.

## Data Availability

The data presented in this study are available on request from the corresponding author.
